# Calmodulin is responsible for Ca^2+^-dependent regulation of TRPA1 Channels

**DOI:** 10.1038/srep45098

**Published:** 2017-03-23

**Authors:** Raquibul Hasan, Alasdair T. S. Leeson-Payne, Jonathan H. Jaggar, Xuming Zhang

**Affiliations:** 1 School of Medicine, Medical Sciences & Nutrition, University of Aberdeen, Foresterhill, Aberdeen AB25 2ZD, United Kingdom; 2 Department of Physiology, University of Tennessee Health Science Center, Memphis, TN 38163, USA; 3 Department of Pharmacology, University of Cambridge, Tennis Court Road, Cambridge, CB2 1PD, United Kingdom; 4 Schcool of Life & Health Sciences, Aston University, Aston triangle, Birmingham B4 7ET, United Kingdom.

## Abstract

TRPA1 is a Ca^2+^-permeable ion channel involved in many sensory disorders such as pain, itch and neuropathy. Notably, the function of TRPA1 depends on Ca^2+^, with low Ca^2+^ potentiating and high Ca^2+^ inactivating TRPA1. However, it remains unknown how Ca^2+^ exerts such contrasting effects. Here, we show that Ca^2+^ regulates TRPA1 through calmodulin, which binds to TRPA1 in a Ca^2+^-dependent manner. Calmodulin binding enhanced TRPA1 sensitivity and Ca^2+^-evoked potentiation of TRPA1 at low Ca^2+^, but inhibited TRPA1 sensitivity and promoted TRPA1 desensitization at high Ca^2+^. Ca^2+^-dependent potentiation and inactivation of TRPA1 were selectively prevented by disrupting the interaction of the carboxy-lobe of calmodulin with a calmodulin-binding domain in the C-terminus of TRPA1. Calmodulin is thus a critical Ca^2+^ sensor enabling TRPA1 to respond to diverse Ca^2+^ signals distinctly.

The survival of animals depends on their ability to detect damaging signals in the environment. TRPA1 is a polymodal damage sensor detecting a wide array of damaging signals including noxious cold, chemical and mechanical stimuli^1^,^2^. TRPA1 has been implicated in various physiological processes and medical conditions, such as chemoreception, pain, itch, inflammation, neuropathy and airway diseases^1^,^3^,^4^,^5^,^6^. TRPA1 has thus become one of the most promising therapeutic targets.

TRPA1 is highly conserved in detecting harmful chemicals across different species^7^. It can be directly activated by many noxious and reactive chemical agonists, such as allyl isothiocyanate (AITC) and cinnamaldehyde^8^,^9^, belonging largely to two main categories: electrophilic and non-electrophilic agonists. Electrophilic agonists, such as AITC, open TRPA1 through covalent modification of cysteine residues on TRPA1^10^,^11^, while non-electrophilic agonists, such as carvacrol, are believed to activate TRPA1 through binding directly to the channel^2^,^12^. In addition to chemosensation, considerable evidence supports a role for TRPA1 in transducing noxious cold^13^,^14^,^15^,^16^,^17^,^18^, though this remains controversial. Furthermore, TRPA1 transduces noxious mechanical stimuli in pathological conditions such as inflammatory pain^2^,^19^,^20^.

Ca^2+^ is a ubiquitous second messenger playing a pivotal role in regulating TRPA1^21^,^22^,^23^. It exerts a bimodal effect on TRPA1. Firstly, Ca^2+^ activates and/or potentiates TRPA1 at low concentrations^21^,^22^,^23^. It is a fundamental mechanism underlying indirect activation of TRPA1 by inflammatory mediators (e.g. histamine) and UV light, both of which act by increasing [Ca^2+^]_i_ after activating phospholipase C (PLC)-coupled receptors^9^,^24^,^25^,^26^. Increased [Ca^2+^]_i_ has also been proposed as a mechanism by which cold activates TRPA1^21^,^27^. Furthermore, Ca^2+^ potentiated TRPA1 response to mechanical stimuli^28^. Ca^2+^-dependent activation and/or potentiation of TRPA1 thus further diversifies the function of TRPA1. Secondly, high Ca^2+^ rapidly inactivates TRPA1 following initial channel activation, a process known as desensitization or tachyphylaxis^12^,^22^,^29^,^30^. This process prevents persistent TRPA1 activation, which otherwise may cause cell and tissue damage^31^.

Despite the central importance of Ca^2+^-dependent regulation of TRPA1, the underlying mechanisms remain elusive. It has been reported that Ca^2+^ activates TRPA1 by directly binding to an EF hand domain in the N-terminus of TRPA1^21^,^23^. However, this finding has not been consistently observed^22^,^23^,^29^,^32^,^33^. Here, we report that Ca^2+^ acts on TRPA1 via Ca^2+^-sensing calmodulin (CaM). CaM binds to TRPA1 depending on Ca^2+^, and is essential for the basal sensitivity of TRPA1. Importantly, CaM binding is sufficient to evoke TRPA1 potentiation and inactivation without changes in Ca^2+^, and this effect can be selectively prevented by disrupting the binding of CaM to TRPA1. Therefore, CaM functions as both a Ca^2+^ sensor and an effector enabling TRPA1 to sense and respond to diverse Ca^2+^ signals distinctly.

## Results

### Ca^2+^ is essential for TRPA1 potentiation, inhibition and desensitization

**Figure 1:**
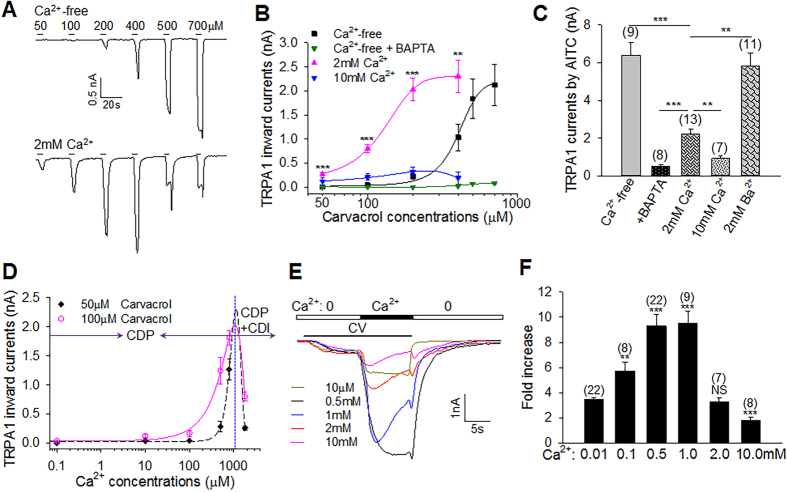
Ca^2+^ potentiates and inactivates TRPA1. (**A**) Representative TRPA1 inward currents elicited by different doses of carvacrol in HEK293 cells expressing TRPA1 in Ca^2+^-free (top) and 2.0 mM Ca^2+^ (bottom). (**B**) Summary of TRPA1 peak currents as a function of carvacrol doses under different concentrations of [Ca^2+^]_e_ from experiments similar to those in (**A**). In BAPTA-AM-treated cells, cells were pre-treated with BAPTA-AM (50 μM) and recorded in Ca^2+^-free. The Ca^2+^-free curve (black), half maximal concentration (EC_50_) = 404.5 μM; Ca^2+^-free with BAPTA-AM curve (green), EC_50_ = 437.5 μM; 2 mM Ca^2+^ curve (pink), EC_50_ = 132.6 μM. All the curves were fitted with Hill equation, except the curve at 10 mM Ca^2+^ fitted with lognormal distribution (blue). All data are mean ± SEM (n = 6–12 for each curve). **P < 0.01; ***P < 0.001, compared to Ca^2+^-free. (**C**) Summary of TRPA1 inward peak currents evoked by AITC (100 μM, 25 s) in different concentrations of Ca^2+^ or Ba^2+^. The number of experiments is given above each bar. (**D**) Ca^2+^-dependent biphasic relationship of TRPA1 currents elicited by carvacrol (50 μM, 100 μM). The dotted vertical blue line indicates Ca^2+^ (∼1 mM) causing the maximal TRPA1 response. Low Ca^2+^ (<1 mM) promotes CDP and high Ca^2+^ (≥1 mM) triggers CDI and CDP. The curves were fitted with the Gaussian function. n = 6–22 for each point. (**E**) Representative traces of TRPA1 responses to carvacrol (CV, 10 s) potentiated by different concentrations of Ca^2+^ as indicated. (**F**) Summary of TRPA1 potentiation induced by different Ca^2+^ in experiments similar to those in (**E**). The number of experiments is given above each bar. Significance was compared to bar 1. All data are mean ± SEM. NS, not significant; **P < 0.01; ***P < 0.001.

Previous studies have shown that low Ca^2+^ concentrations potentiate TRPA1 while high Ca^2+^ concentrations desensitize TRPA1^22^,^23^,^30^. To investigate the role of Ca^2+^ in the modulation of TRPA1, we recorded TRPA1 currents from HEK293 cells expressing TRPA1 elicited by the non-electrophilic agonist carvacrol. We found that peak TRPA1 currents increased progressively with increased concentrations of carvacrol without appreciable desensitization in the absence of [Ca^2+^]_e_ (Ca^2+^-free, 0 [Ca^2+^]_e_ + 5 mM EGTA) (Fig. 1A, top panel). Interestingly, when both [Ca^2+^]_e_ and [Ca^2+^]_i_ were chelated (Ca^2+^-free + BAPTA-AM), negligible TRPA1 currents were induced across all doses of carvacrol (Fig. 1B, green curve), suggesting that basal [Ca^2+^]_i_ is essential for TRPA1 activation and/or potentiation. We then investigated the effect of [Ca^2+^]_e_ on TRPA1 in the presence of 2 mM [Ca^2+^]_e_, TRPA1 exhibited substantially larger currents and much higher sensitivity at lower doses of carvacrol (<400 μM), leading to a marked leftward shift in the dose-response curve (Fig. 1A and B). Higher concentrations of carvacrol (>500 μM) elicited much smaller currents, apparently due to prominent TRPA1 desensitization (Fig. 1A). Similar effects were also observed with the electrophilic agonist AITC (Fig. 1C). These data are consistent with the finding that [Ca^2+^]_e_ promotes both TRPA1 activation and desensitization^22^. In addition, we show that Ca^2+^ influences the basal sensitivity or responsiveness of TRPA1 to channel activators.

Intriguingly, a further increase of [Ca^2+^]_e_ to 10 mM also inhibited TRPA1 responses to both carvacrol and AITC to a similar degree as removing [Ca^2+^]_e_ and [Ca^2+^]_i_ (Fig. 1B and C), indicating that high [Ca^2+^]_e_ also inhibits the basal sensitivity of TRPA1, likely due to an elevation in [Ca^2+^]_i_ caused by the basal entry of [Ca^2+^]_e_, leading to channel inhibition. Therefore, Ca^2+^ regulates multiple aspects of TRPA1 function including potentiation/activation, inhibition and desensitization. For clarity, both TRPA1 inhibition and desensitization are termed inactivation, though it is unknown whether the two processes employ the same mechanism.

To define the [Ca^2+^]_e_ range that triggers potentiation and inactivation, respectively, we plotted TRPA1 currents elicited by carvacrol as a function of different concentrations of [Ca^2+^]_e_, yielding a biphasic response curve (Fig. 1D). Based on this curve, [Ca^2+^]_e_ evoking the maximal TRPA1 response was estimated to be around 1 mM (Fig. 1D). Ca^2+^ below 1 mM is thus defined as low Ca^2+^. Within this range, Ca^2+^ mainly promoted TRPA1 activation with little inhibition or desensitization (Fig. 1D), a process manifesting Ca^2+^-dependent potentiation (CDP). By contrast, the inhibition and/or desensitization of TRPA1 became more and more prominent with further increases in Ca^2+^ at the high Ca^2+^ range (i.e. >1 mM), resulting in gradually reduced TRPA1 responses, a process reflecting Ca^2+^-dependent inactivation (CDI), in addition to CDP (Fig. 1D, also see below).

We next examined TRPA1 potentiation induced by low and high Ca^2+^, respectively, in real-time. Figure 1E shows that carvacrol elicited stable inward TRPA1 currents in nominal 0 Ca^2+^. The currents were then rapidly potentiated shortly after Ca^2+^ was perfused. Of note, Ca^2+^-induced TRPA1 potentiation progressively enhanced with increases in perfused Ca^2+^, with a peak potentiation at around 1 mM Ca^2+^ (Fig. 1E and F), consistent with predicated peak TRPA1 response at 1 mM Ca^2+^ in Fig. 1D. Notably, peak TRPA1 currents were not desensitized in the presence of carvacrol at low Ca^2+^ (Fig. 1E), showing that low Ca^2+^ induces a pure CDP process. By contrast, high Ca^2+^-induced peak TRPA1 currents rapidly reduced following initial potentiation, a process known as acute desensitization (Fig. 1E), leading to a gradual reduction in the overall CDP. CDP at high Ca^2+^ is thus a consequence of counteracting actions of concurrent CDP and CDI. Collectively, Ca^2+^ is a key player in multiple TRPA1 functions including the basal sensitivity, CDP and CDI.

### TRPA1 binds to CaM depending on Ca^2+^

We next investigated how TRPA1 senses and responds to different Ca^2+^ levels. Previous studies did not consistently support the idea that direct binding of Ca^2+^ to TRPA1 mediates the Ca^2+^ effects (see introduction), we wondered whether Ca^2+^ act through an intermediate protein, which is not only sensitive to Ca^2+^, but also binds to TRPA1, forming a Ca^2+^-sensitive channel complex. CaM is such a candidate with remarkable Ca^2+^-sensing capability. Indeed, CaM mediates Ca^2+^ gating of many ion channels, such as TRP, SK, Na^+^ and Ca^2+^ channels^34^,^35^,^36^,^37^,^38^,^39^.

**Figure 2:**
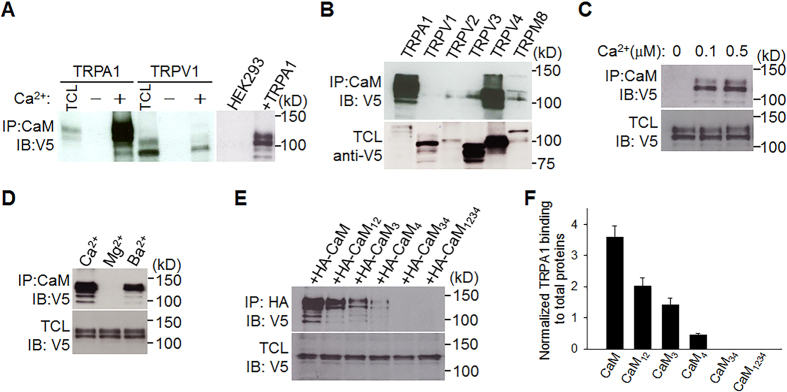
CaM binds to TRPA1 depending on Ca^2+^. (**A**) HEK293 cell lysates expressing TRPA1-V5 or TRPV1-V5 (left panel) or non-transfected HEK293 cell lysates (lane 1 on the right panel) were pulled down by CaM-agarose with or without Ca^2+^ (1 mM) followed by detection with anti-V5. Total cell lysate (TCL) shows the expression of TRPA1 and TRPV1. Molecular weight is shown on the right, similar to all other blots. (**B**) Different thermo-TRP ion channels were pulled down by CaM-agarose in 2 mM Ca^2+^. The bottom blot shows the expression of individual TRP channels in total cell lysate (TCL). Multiple bands of TRP channels are caused by channel glycosylation^56^. The relative binding of TRPA1, TRPV1, 2, 3, 4 and TRPM8 to CaM (normalized by their total input) is 22.5, 0.3, 0.8, 0.2, 1.4 and 0.9, respectively. (**C**,**D**) CaM pull down assay shows the interaction of CaM with TRPA1 in nanomolar ranges of Ca^2+^ (**C**) and in different cations (1 mM each) (**D**). The bottom blots show equal expression of TRPA1 in different groups. (**E**) HA-tagged CaM or related mutants co-expressed with TRPA1-V5 in HEK293 cells as indicated, was immuno-precipitated (IP) by anti-HA in 2 mM Ca^2+^ followed by detection with anti-V5 (top blot). The bottom blot shows total input TRPA1 proteins. (**F**) Summary of relative TRPA1 binding to CaM (normalized by total TRPA1 proteins shown at the bottom blot in E) from similar experiments to those in E (n = 3).

To test this hypothesis, we first examined whether CaM binds to TRPA1 using CaM pull-down assay. TRPV1 is known to be regulated by CaM^36^, and was thus used as a positive control. We found that CaM bound to much more TRPA1 than to TRPV1 in the presence of Ca^2+^. The binding was, however, absent without either Ca^2+^ or TRPA1/TRPV1 co-expression (Fig. 2A). Notably, the most potent binding to CaM was observed with TRPA1 among thermo-TRP channels (Fig. 2B), suggesting a high binding affinity of CaM for TRPA1. Indeed, TRPA1-CaM binding can even be triggered by nanomolar ranges of Ca^2+^ (Fig. 2C), implying that TRPA1 binds to CaM in the resting [Ca^2+^]_i_ (∼100 nM). Furthermore, despite a much lower affinity of CaM for Ba^2+^ over Ca^2+^ ions^40^, Ba^2+^ elicited substantial TRPA1-CaM binding, albeit reduced when compared to Ca^2+^ (Fig. 2D), further supporting a high binding affinity between TRPA1 and CaM. Consistent with Ba^2+^-induced TRPA1-CaM binding, Ba^2+^ was capable of regulating TRPA1 (see below). However, Mg^2+^ did not induce significant TRPA1-CaM binding (Fig. 2D), and accordingly did not modulate TRPA1 (see below).

In support of CaM pull-down assay, TRPA1 was found to bind to CaM in a co-immunoprecipitation experiment (Fig. 2E). CaM contains two different lobes (N- and C-lobe) and each lobe contains two EF hand domains responsible for Ca^2+^ binding. CaM C-lobe exhibits a higher binding affinity for Ca^2+^ (*Kd*, 10^−7^ M) than N-lobe (*Kd*, 10^−6^ M)^41^. The binding of Ca^2+^ to CaM can thus be selectively disrupted by mutating two different lobes^42^. There was about a 40% reduction in TRPA1-CaM binding after selectively preventing Ca^2+^ binding to N-lobe by mutating the first two Ca^2+^ binding sites (EF_12_) on N-lobe (CaM_12_ mutant) (Fig. 2E and F). Mutating the third (EF_3_) and the fourth Ca^2+^ binding sites (EF_4_) on C-lobe (CaM_3_, CaM_4_ mutants) caused a 60% and an 87% reduction in TRPA1-CaM binding, respectively (Fig. 2E and F). TRPA1-CaM binding was, however, completely abolished by mutating both Ca^2+^ binding sites on C-lobe (CaM_34_ mutant), or by Ca^2+^-insensitive CaM_1234_ in which all four Ca^2+^-binding sites are mutated^34^ (Fig. 2E and F). Therefore, binding of CaM to TRPA1 critically depends on Ca^2+^ loading of CaM, and different Ca^2+^-binding sites on CaM play distinct roles in TRPA1 binding: CaM C-lobe is more important in triggering TRPA1 binding, while N-lobe may only play an auxiliary role in the binding process. Even within CaM C-lobe, the role of EF_3_ and EF_4_ is not identical, with EF_4_ having a higher capability of triggering TRPA1 binding than EF_3_.

### CaM binds to a non-canonical CaM-binding domain (CaMBD) in TRPA1

**Figure 3:**
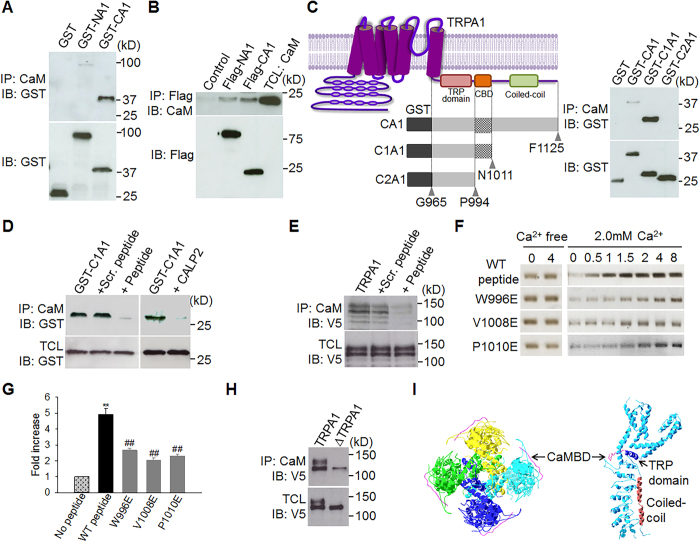
Identifying a CaM-binding domain (CaMBD) in the C-terminus of TRPA1. (**A**) The N- and C-termini of TRPA1 coupled to GST (GST-NA1, GST-CA1) were purified and then incubated with CaM-agarose, followed by detection with ant-GST (top blot). The bottom blot shows the expression of GST-coupled protein fragments. (**B**) The N and C termini of TRPA1 coupled to Flag (Flag-NA1, -CA1) were purified by Flag-agarose and then incubated with pure CaM. Bound CaM was detected by anti-CaM (top blot). The same blot was stripped and reprobed with ant-Flag (bottom blot). (**C**) A schematic diagram (top) shows the CaM-binding domain (CBD), TRP domain and coiled coil domain in the C-terminus of TRPA1. The corresponding truncated GST-CA1 fragments as illustrated were purified and used for CaM pull down assay on the right (top blot). The bottom blot on the right shows the expression of purified GST-coupled TRPA1 fragments. (**D**) The binding of purified GST-C1A1 fragment to CaM was blocked by the CaMBD peptide and by the CaM antagonistic peptide CALP2, but not by a scrambled CaMBD peptide. 200 μM was used for all the peptides. (**E**) The binding of TRPA1-V5 to CaM in a CaM-pull down assay was blocked by the CaMBD peptide (peptide), but not by a scrambled one (Scr. peptide). (**F**) Silver staining of CaM complexed with the indicated molar ratios of peptide: CaM with or without Ca^2+^. WT peptide denotes the CaMBD peptide. (**G**) Summary of CaM intensity at the peptide: CaM ratio of 4 relative to that at the ratio of 0 for different peptides. Data are mean ± SEM (n = 3). **P < 0.01; ^##^*P* < 0.01 compared to Bar 2. (**H**) Binding of CaM to TRPA1 and CaMBD-deleted TRPA1 (ΔTRPA1) revealed by a CaM pull down assay. The bottom blot shows the total input TRPA1 protein (TCL). (**I**) Ribbon diagrams depicting the identified CaMBD (pink) in the overall structure of TRPA1 (PDB: 3J9P) (left, bottom view; right, side view). Four individual TRPA1 subunits in homomeric TRPA1 channel complex (left) is shown in yellow, cyan, blue and green, respectively.

CaM binds to a variety of molecular targets through several classes of CaMBD such as the “IQ” motif in Ca^2+^ channels^43^,^44^,^45^. However, TRPA1 does not contain any known canonical CaMBDs. To delineate a CaMBD on TRPA1, the cytoplasmic N- and C-terminal fragments of TRPA1 coupled to the GST tag were first purified and then used for CaM pull-down assay. CaM bound prominently to the C-terminus of TRPA1, but not to GST, though there was a negligible binding to the N-terminus (Fig. 3A). Similar results were also obtained with Flag pull-down assay in which purified Flag-coupled TRPA1 cytoplasmic tails were used to pull down pure CaM (Fig. 3B). To further narrow down the binding, we truncated the C-terminus of TRPA1 progressively. Deleting the distal 114 amino acids (AA) (R1012-F1125) enhanced CaM binding, but a further deletion of 17 residues (L995-N1011) completely eliminated CaM binding (Fig. 3C), suggesting that the 17AA is a key CaMBD.

To validate whether the 17AA is a *bona fide* CaMBD, we synthesized a peptide identical in sequence to the 17AA. As anticipated, the binding of CaM to the C-terminus of TRPA1 was abrogated by incorporating the peptide in CaM pull-down assay (Fig. 3D). A scrambled peptide was, however, ineffective (Fig. 3D). As a positive control, the binding was also abolished by the CaM antagonistic peptide CALP2 (Fig. 3D)^46^. The specificity of the CaMBD peptide was further tested on full-length TRPA1. Consistently, TRPA1-CaM binding was almost abolished by the CaMBD peptide, but not by the scrambled peptide (Fig. 3E).

To verify whether CaM binds directly to the CaMBD peptide forming a CaM-peptide complex, we incubated the peptide with pure CaM in different molar ratios and then resolved protein complexes on non-denaturing PAGE gels followed by silver staining for visualization. CaM did not exhibit a mobility shift after incubation with the peptide (Fig. 3F, top panel), presumably the peptide is not large enough (17aa) to alter CaM mobility. But intriguingly, the intensity of CaM gradually enhanced with increasing ratios of peptide to CaM in the presence of Ca^2+^. The effect was abolished by removing Ca^2+^ (Fig. 3F and G), suggesting the formation of a Ca^2+^-dependent CaM-peptide complex, which contains more amino acid side chains for binding to silver ions than pure CaM, resulting in enhanced silver stain. These experiments conclusively demonstrate that the 17 AA in the C-terminus of TRPA1 is a *bona fide* CaMBD and that CaM binds directly to TRPA1.

We next deleted the CaMBD from TRPA1. As expected, CaM binding was dramatically reduced in CaMBD-lacking TRPA1 (ΔTRPA1), though incompletely (Fig. 3H), suggesting the presence of other minor CaM binding regions elsewhere in TRPA1, likely in the N terminus.

Of note, the CaMBD is rich in hydrophobic residues, consistent with the binding preference of CaM^44^. Interestingly, based on the recently resolved TRPA1 structure^47^, the CaMBD corresponds precisely to the β-strand domain with unknown function flanked by the TRP-like domain and coiled-coil domain in the C-terminus of TRPA1 (Fig. 3I), two important structural domains critical for modulating TRPA1 gating^47^. Notably, the CaMBD is freely exposed in the periphery of TRPA1 structure (Fig. 3I)^47^, making this domain physically accessible to CaM.

### CaM is essential for TRPA1 CDP

**Figure 4:**
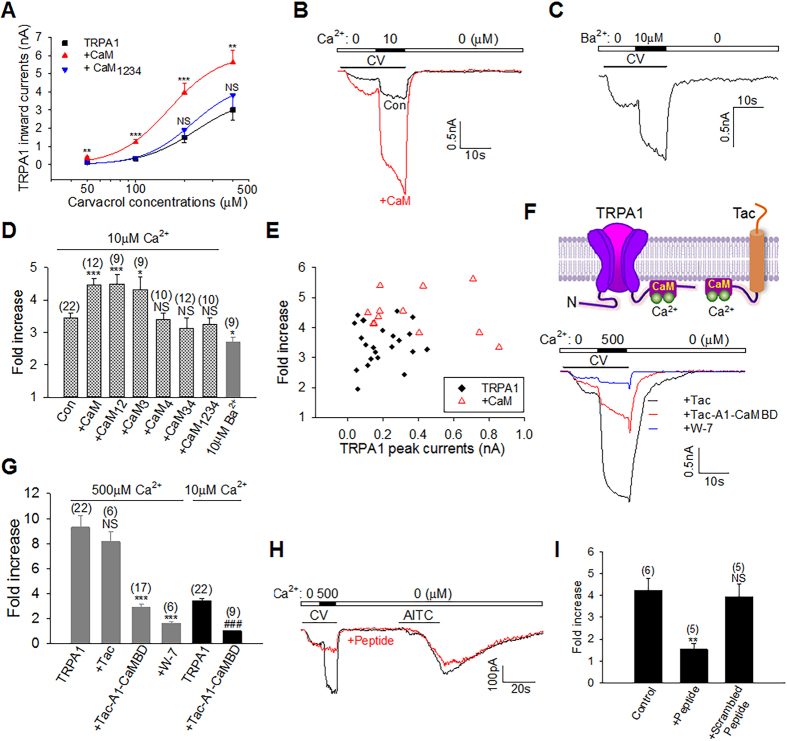
CaM enhances TRPA1 sensitivity and is essential for TRPA1 CDP in low Ca^2+^. (**A**) Dose-response relationship between TRPA1 peak currents and carvacrol concentrations in HEK293 cells expressing TRPA1 or together with CaM mutants in nominal 0 Ca^2+^ (n = 6–10 for each curve). (**B**) Example TRPA1 currents evoked by carvacrol (CV, 100 μM) potentiated by Ca^2+^ (10 μM) with (red) or without CaM. (**C**) A typical TRPA1 current evoked by carvacrol (CV, 100 μM) was potentiated by Ba^2+^ (10 μM). (**D**) A summary of TRPA1 potentiation caused by 10 μM Ca^2+^ or Ba^2+^ in experiments similar to those in (**B**,**C**) in the presence of different CaM mutants. The number of experiments is given above each bar. (**E**) Scatter plots of Ca^2+^-induced TRPA1 potentiation fold as a function of initial TRPA1 peak currents from the same cells used in (**B** and **D**). Cells expressing TRPA1 only (black diamond) correspond to the control group in (**D**). (**F**) Schematic diagram shows the binding of Ca^2+^-CaM to the C terminus of TRPA1 and to the chimeric Tac-A1-CaMBD (top). Underneath are representative TRPA1 currents elicited by carvacrol (CV, 100 μM) potentiated by Ca^2+^ in cells co-expressing Tac (black) or Tac-A1-CaMBD (red) or perfused with W-7 (100 μM) (blue). (**G**) Collective results of TRPA1 potentiation induced by Ca^2+^ (10 μM, 500 μM) from experiments similar to those in (**F**). The number of experiments is shown above each bar. (**H**,**I**) Example (**H**) and summary (**I**) of Ca^2+^-induced potentiation of TRPA1 currents evoked by carvacrol (CV, 100 μM) in DRG neurons. AITC (100 μM, 25 s) was applied at the end. The potentiation was prevented by the CaMBD peptide (200 μM) (**H**,**I**), but not by the scrambled peptide (**I**). The number of experiments is indicated above each bar. All data are mean ± SEM. NS, not significant; **P < 0.01; ***P < 0.001; ^###^*P* < 0.001 compared to bar 5.

To test a possible role for CaM in regulating TRPA1, we first investigated whether CaM potentiates TRPA1, as does low Ca^2+^. Indeed, CaM over-expression potently increased TRPA1 responses to all doses of carvacrol in nominal 0 [Ca^2+^]_e_ (Fig. 4A), suggesting that TRPA1 is not fully occupied by endogenous CaM under the resting condition. The effect of CaM was, however, absent in Ca^2+^-free CaM_1234_, which was also deficient for TRPA1 binding (Figs 2E and 4A). These data demonstrate that CaM binding is sufficient to potentiate the basal sensitivity of TRPA1 even without changes in [Ca^2+^]_e_. It is likely that the basal [Ca^2+^]_i_ (∼100 nM) is sufficient to trigger enhanced TRPA1-CaM binding in the presence of excess CaM.

We then examined whether CaM mediates CDP. To isolate CDP from CDI and to avoid the mutual interference of the two processes, we used the Ca^2+^ range between 10 μM and submaximal 0.5 mM that elicits no appreciable desensitization. TRPA1 current evoked by carvacrol was rapidly potentiated by 10 μM Ca^2+^ (Fig. 4B). A similar potentiation was also observed with Ba^2+^, though to a less degree (Fig. 4C and D). Ca^2+^-induced TRPA1 potentiation was significantly enhanced when cells overexpressed CaM or CaM_12_ or CaM_3_ (Fig. 4B and D), consistent with their ability to bind to TRPA1 (Fig. 2E). Such enhancement was absent in cells expressing CaM_4_, or CaM_34_ or CaM_1234_ mutants (Fig. 4D), all of which exhibited little (CaM_4_) or no binding (CaM_34_, CaM_1234_) to TRPA1 (Fig. 2E), suggesting that CaM binding is critical for TRPA1 CDP and that EF_4_ on CaM C-lobe is indispensable for this process. Enhanced CDP may be caused by a larger Ca^2+^ influx due to increased initial TRPA1 responses evoked by carvacrol in the presence of overexpressed CaM. However, there were no positive correlations between the initial peak current amplitudes and CDP (r = 0.044, Fig. 4E), suggesting that enhanced CDP is not due to different [Ca^2+^]_e_ entry. Taken together, increases in either Ca^2+^ or CaM can elicit CDP. Ca^2+^ and CaM thus act cooperatively to potentiate TRPA1 under low Ca^2+^.

To determine whether CDP could be prevented by disrupting TRPA1-CaM binding, we took advantage of the potent binding between CaM and TRPA1 CaMBD, and substituted the CaMBD for the cytoplasmic tail in Tac antigen, an α-subunit of interleukin-2 receptor with a single membrane-spanning domain^48^, to produce a Tac-A1-CaMBD chimera (Fig. 4F). We then co-expressed Tac-A1-CaMBD to sequester endogenous CaM. Co-expressed Tac-A1-CaMBD markedly reduced CDP induced by 0.5 mM Ca^2+^, and completely abolished CDP evoked by 10 μM Ca^2+^ (Fig. 4F and G). In contrast, CDP was not significantly affected by Tac co-expression (Fig. 4F and G). Similarly, TRPA1 CDP was also blocked by the CaM antagonist W-7 (Fig. 4F and G), validating an essential role for CaM in eliciting TRPA1 CDP. TRPA1 CDP was also observed in DRG neurons (Fig. 4H). Importantly, CDP was significantly prevented by including the CaMBD peptide in the pipette, while a scrambled peptide was without effect (Fig. 4H and I). Collectively, these experiments demonstrate a critical role for CaM in governing TRPA1 sensitivity and CDP.

### CaM is critical for TRPA1 CDI

**Figure 5:**
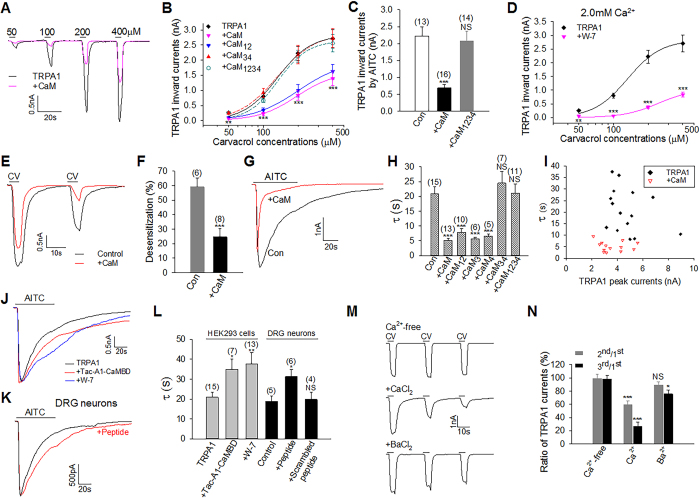
CaM inhibits TRPA1 sensitivity and is critical for TRPA1 desensitization in high Ca^2+^. (**A**) Typical TRPA1 currents elicited by different doses of carvacrol from HEK293 cells expressing TRPA1 or with CaM. (**B**) TRPA1 inward currents as a function of cavacrol concentrations in HEK293 cells co-expressing TRPA1 and CaM mutants (n = 6–15 for each curve). (**C**) Bar summary of TRPA1 currents elicited by AITC (100 μM) in 2.0 mM Ca^2+^ in HEK cells expressing TRPA1. The number of experiments is shown above each bar. (**D**) Dose-response curve of TRPA1 from experiments similar to those in (**B**), but perfused with W-7 (100 uM). (**E**) Representative TRPA1 currents evoked by two consecutive pulses of carvacrol (CV, 400 μM) in HEK cells expressing TRPA1 or with CaM (red). (**F**) Summary of percentage of TRPA1 desensitization from experiments similar to those in (**E**). (**G**) Representative currents evoked by AITC (1 mM) from cells expressing TRPA1 (Con) or with CaM. (**H**) Bar summary of time constant (τ) of TRPA1 desensitization caused by CaM mutants from experiments similar to those in (**G**). (**I**) Scatter plots of Ca^2+^-induced desensitization rate (τ) of TRPA1 as a function of initial peak currents from the same cells used in (**G** and **H**). Cells expressing TRPA1 only correspond to the control group in (**H**). (**J**) Example TRPA1 currents elicited by AITC (1 mM) in cells co-expressing Tac-A1-CaMBD or perfused with W-7 (100 μM) in 2 mM Ca^2+^. Peak currents are scaled down 1.3-fold for Tac-A1-CaMBD and up 1.7-fold for W-7 for kinetic comparison. (**K**) Currents evoked by AITC (1 mM) in DRG neurons with or without the CaMBD peptide (200 μM) in patch pipette. The peak current for the peptide is scaled up 1.2-fold for kinetic comparison. (**L**) Collective results of time constant of TRPA1 desensitization from similar experiments to those in (**J** and **K**). (**M**) Representative TRPA1 currents induced by carvacrol (CV, 400 μM) in Ca^2+^-free, Ca^2+^ (2 mM) and Ba^2+^ (2 mM). (**N**) Summary of ratio of the 2^nd^/3^rd^ peak currents to the 1^st^ peak currents from experiments similar to those in (**M**) (n = 6–11 each). All data are mean ± SEM. NS, not significant; *P < 0.01; **P < 0.001; ***P < 0.001.

To investigate a role of CaM in regulating TRPA1 CDI, we first examined whether CaM inhibits TRPA1 in high Ca^2+^ (>1 mM). Indeed in 2 mM Ca^2+^, TRPA1 currents evoked by all doses of carvacrol were robustly inhibited by over-expressed CaM (Fig. 5A and B), an effect analogous to that induced by higher 10 mM Ca^2+^ (Fig. 1B). Thus, increases in either Ca^2+^ or CaM inhibit TRPA1 in the high Ca^2+^ range. The inhibitory effect was also observed with the N-lobe mutant CaM_12_, but not with the C-lobe mutant CaM_34_ or Ca^2+^-insensitive CaM_1234_ (Fig. 5B), suggesting a critical role for CaM C-lobe in this process. Similar effects were also observed with another TRPA1 agonist AITC (Fig. 5C). In contrast to a previous report^21^, the sensitivity of TRPA1 was also inhibited by blocking endogenous CaM with the CaM antagonist W-7 (Fig. 5D), further supporting the idea that CaM is essential for the basal sensitivity of TRPA1.

We then investigated whether CaM is involved in TRPA1 desensitization. To this end, TRPA1 was activated by consecutive pulses of carvacrol. Peak TRPA1 current was typically reduced in the second activation resulting in tachyphylaxis (Fig. 5E). CaM overexpression promoted TRPA1 tachyphylaxis (Fig. 5E and F). To measure acute desensitization, TRPA1 was maximally activated by AITC to allow desensitization to fully evolve following initial channel activation (Fig. 5G). Remarkably, CaM overexpression accelerated the desensitization rate of TRPA1, leading to a marked reduction in the time constant of desensitization (Fig. 5G and H), despite overall smaller peak currents with CaM (Fig. 5G and I). The accelerated desensitization of TRPA1 was not affected by CaM_12_, CaM_3_ and CaM_4_, all of which retained TRPA1 binding, but was absent in the C-lobe mutant CaM_34_ and CaM_1234_, both of which were deficient for TRPA1 binding (Figs 2E and 5H), suggesting that CaM binding is also crucial for inducing TRPA1 desensitization and that EF_34_ in CaM C-lobe is critical in this process. Taken together, CaM inhibits TRPA1 sensitivity in the basal state and promotes TRPA1 desensitization in the activation state of the channel in high Ca^2+^, and both processes require CaM C-lobe.

To investigate whether TRPA1 desensitization could be prevented by disrupting TRPA1-CaM binding, we used the CaM chelator Tac-A1-CaMBD and the CaMBD peptide. In HEK293 cells expressing TRPA1, co-expression of Tac-A1-CaMBD significantly reduced the desensitization rate of TRPA1 induced by 2 mM Ca^2+^ (Fig. 5J and L). An analogous effect was also observed with the CaM antagonist W-7 (Fig. 5J and L). In native DRG neurons, Currents induced by the TRPA1 agonist AITC underwent similar rapid desensitization (Fig. 5K and L). The acute desensitization was significantly prevented by including in the pipette the CaMBD peptide, but not by a scrambled peptide (Fig. 5L), further demonstrating that CaM is critical for TRPA1 desensitization. As an alternative approach, we used Ba^2+^, which elicited a reduced TRPA1-CaM binding compared to Ca^2+^ (Fig. 2D). Accordingly, Ba^2+^ prevented desensitization induced by consecutive pulses of carvacrol (Fig. 5M and N). These experiments further demonstrate that CaM binding is required for TRPA1 desensitization.

### CaM binding sites responsible for TRPA1 CDP and CDI

**Figure 6:**
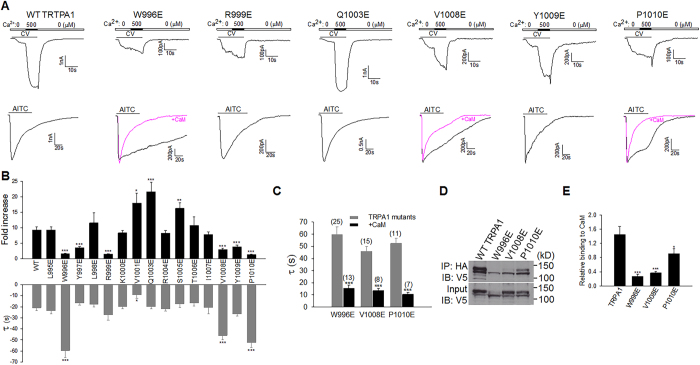
Ca^2+^/CaM effector sites on TRPA1 mediating CDP and CDI. (**A**) Representative Ca^2+^-induced potentiation (top panels) and desensitization current traces (bottom panels) of different TRPA1 mutants. For potentiation, TRPA1 currents were induced by carvacrol (CV, 50 μM; 100 μM for W996E). For desensitization, 1 mM AITC (60 s) were applied. Blunted desensitization of the W996E, V1008E and P1010E mutants was rescued by overexpressing CaM (pink). (**B**) Summary of potentiation fold and time constant (τ) of desensitization of TRPA1 mutants from experiments similar to those in (**A**). All data are mean ± SEM. NS, not significant; *P < 0.05; **P < 0.01; ***P < 0.001 compared with wild type (WT) TRPA1 (n = 5–25 each). (**C**) Summary of rescue of blunted desensitization of TRPA1 mutants by CaM. The number of experiments is shown above each bar. Data are mean ± SEM. ***P < 0.001. (**D**) Co-immunoprecipitation (IP) of HA-CaM with TRPA1-V5 mutants in 2 mM Ca^2+^. TRPV1 mutants were first purified using Ni-NTA beads, and equal amount of different total TRPA1 mutants was used for Co-IP (bottom blot). (**E**) Summary of relative binding of different TRPA1 mutants to CaM normalized by total input proteins from experiments similar to those in (**D**). All error bars are mean ± SEM. *P < 0.05; ***P < 0.001 (n = 3).

To identify CaM effector sites on TRPA1 responsible for CDP and CDI, we mutated 16 residues on CaMBD individually to the negatively charged glutamic acid, in an attempt to disrupt TRPA1-CaM interaction. To investigate the effect of these mutants on CDP, the mutated channels were activated using lower dose of carvacrol in order to avoid saturating TRPA1 mutants based on their dose-response curves (data not shown). TRPA1 CDP was abolished by mutating W996, R999, and P1010 (Fig. 6A and B). A pronounced deficit in CDP was also observed in Y997E, V1008E and Y1009E TRPA1 (Fig. 6A and B). Interestingly, three of these mutants (W996E, V1008E and P1010E) also exhibited significant impairment in acute desensitization induced by AITC (Fig. 6A and B). The three sites are thus critical for transducing both CDP and CDI. In contrast, three other mutants (i.e. Y997E, R999E, and Y1009E) exhibited selective deficit in CDP without an impairment in CDI (Fig. 6A and B). Notably, impaired desensitization in W996E, V1008E and P1010E was rescued by over-expressing CaM (Fig. 6A and C), showing that impaired desensitization is due to diminished CaM binding and is not intrinsic to these mutants. On the other hand, V1001E exhibited an enhanced potentiation and accelerated desensitization (Fig. 6B). A significant enhancement in CDP was also observed in Q1003E and S1005E (Fig. 6B). Presumably, mutating these sites caused over-compensation of CaM binding to other functional sites such as W996. Neither potentiation nor desensitization was affected in other mutants (Fig. 6C).

We also tested whether CDP and CDI are affected in ΔTRPA1 in which CaMBD has been deleted, but found that ΔTRPA1 was non-functional (data not shown). The lack of function in ΔTRPA1 could be due either to impaired CaM binding or to structural disturbance. Of note, tryptophan is a conserved residue targeted by CaM among many other CaM effectors, such as CaMKII^49^, underscoring a pivotal role for W996 in governing Ca^2+^ gating of TRPA1. Taken together, these results demonstrate that TRPA1 CDP and CDI are mediated by overlapping but distinct sets of effector sites on the CaMBD of TRPA1.

We finally demonstrated whether CaM binding is disrupted in the TRPA1 mutants with defective CDP/CDI. In silver staining of CaM-peptide complex, enhanced CaM staining due to bound CaMBD peptide was significantly diminished by mutating either W996, or V1008 or P1010 (Fig. 3F and G), suggesting that CaM-peptide binding is disrupted in these mutants. A further experiment from co-immunoprecipitation also validated that CaM binding was significantly impaired in the W996E, V1008E and P1010E mutants (Fig. 6D and E).

## Discussion

Ca^2+^ exerts multiple and opposing effects on TRPA1. However, it has been a mystery how TRPA1 senses different Ca^2+^ levels (Ca^2+^ sensor) and translates Ca^2+^ signals into the gating of the channel (Ca^2+^ effector), leading to opposing effects. In this study we have found that Ca^2+^ gating of TRPA1 depends on CaM, which binds to TRPA1, forming a Ca^2+^-sensing channel complex. In the basal state, CaM either enhances (low Ca^2+^) or inhibits (high Ca^2+^) TRPA1 sensitivity. In the activated state, CaM promotes either TRPA1 activation (low Ca^2+^) or desensitization (high Ca^2+^). These effects were prevented by either mutating the Ca^2+^-binding sites on CaM or by mutating the CaM binding sites on TRPA1. CaM thus acts as a Ca^2+^ sensor and an effector responsible for regulating TRPA1 sensitivity and activation in both the basal state and activation state.

Unexpectedly, the multiple Ca^2+^ effects were mediated by CaM C-lobe, without a significant role for N-lobe in these processes. The two lobes of CaM are thus not the source that drives opposing Ca^2+^ effects on TRPA1. This is in contrast to CaV_2.1_ and TMEM16 chloride channels that employ the C-lobe and N-lobe of CaM to transduce channel facilitation and inactivation, respectively^45^,^50^. Interestingly, EF_3_ and EF_4_ in the C-lobe triggered different degrees of TRPA1 binding (Fig. 2E), suggesting that EF_3_ and EF_4_ have different Ca^2+^-binding capabilities and maybe the origin responsible for detecting different Ca^2+^ levels for TRPA1. Consistently, EF_3_ and EF_4_ participate in different aspects of TRPA1 regulation. Specifically, EF_3_ is only involved in TRPA1 CDI, but EF_4_ participates in both TRPA1 CDP and CDI. In keeping with distinct roles of EF_3_ and EF_4_ in TRPA1 regulation, some CaM effector sites on TRPA1 mediated only CDP, while others transduced both CDP and CDI (Fig. 6B). It is thus conceivable that TRPA1 CDP and CDI may be mediated by two different forms of interactions between CaM C-lobe and CaM effector sites on TRPA1, with one form of interaction promoting CDP and another favouring CDI. This possibility could arise from different binding stoichiometries between TRPA1 and CaM.

It is noteworthy that over-expressed CaM can still exert an additional effect on TRPA1 CDP/CDI, supporting that TRPA1 is far from saturated by endogenous CaM. TRPA1-CaM binding can even be triggered by nanomolar ranges of Ca^2+^ (Fig. 2C), suggesting that basal [Ca^2+^]_i_ is sufficient to cause a preassociation of CaM with TRPA1, which may explain why TRPA1 still exhibited Ca^2+^ sensitivity in inside-out excised patches^21^,^22^,^23^, a patch configuration in which all associated intracellular molecules (e.g. CaM) are assumed to have been lost. It is very likely that residual CaM remains associated with excised TRPA1 channels due to, for example, local Ca^2+^ microdomains^51^, which could prevent the complete loss of associated CaM. However, Ca^2+^-free CaM_1234_ (apoCaM) did not pre-associate with TRPA1, in contrast to other ion channels, such as L-type Ca^2+^ channels^43^,^52^. The lack of binding of apoCaM with TRPA1 explains why CaM_1234_ had no dominant-negative action on TRPA1, which is the main evidence leading to the exclusion of a possible role for CaM in TRPA1 regulation in a previous study^21^.

Our results support that the binding of CaM C-lobe is critical for Ca^2+^-dependent regulation of TRPA1. Indeed, all the TRPA1-CaM binding can be eliminated by the C-lobe mutant CaM_34_ (Fig. 2E). In contrast, no associated deficits in TRPA1 modulation were observed in the N-lobe mutant CaM_12_, even though CaM_12_ caused a 40% reduction in TRPA1 binding, suggesting that N-lobe may only play an accessory role in the binding process. It is possible that once loaded with Ca^2+^, CaM C-lobe acts as both a tether anchoring CaM to CaMBD in TRPA1 and an effector transmitting Ca^2+^-dependent channel gating, whereas Ca^2+^-loaded N-lobe may trigger the binding of CaM to other regions in TRPA1, such as the N terminus, acting as a second tether but without influencing channel gating. This possibility is supported by the evidence that there was a weak binding between CaM and the N terminus of TRPA1 (Fig. 3A and B) and that deleting CaMBD from C-terminal TRPA1 did not completely abolish CaM binding (Fig. 3H). However, it remains to be tested whether there is a second CaMBD in the N terminus of TRPA1 contributing to Ca^2+^-dependent modulation of TRPA1.

In this case, a proximal N-terminal ankyrin repeat (AR) domain in TRPA1 has been implicated as a critical region for mediating TRPA1 desensitization^29^. However, we did not find the binding of CaM to the AR domain (data not shown). The AR domain is thus unlikely to be a second CaMBD. A most likely second CaMBD seems to lie in the region nearby the linker domain and/or pre-S1 helix in the N terminus of TRPA1. These regions are structurally in close proximity to the C-terminal CaMBD (Fig. 3I)^47^, and could cooperate with each other to modulate TRPA1 CDP and CDI.

The C-terminal CaMBD of TRPA1 is adjacent to the ion permeation pathway allowing CaM to detect rapid Ca^2+^ oscillations, agreeing with previous studies demonstrating that [Ca^2+^]_e_ regulates TRPA1 through binding to a channel site that needs to be very close to the channel pore^22^,^30^. Notably, the identified six CaM effector sites on TRPA1 are very close either to the TRP domain or to the coiled-coil domain (Fig. 3I), two important structural domains implicated in TRPA1 gating. It is conceivable that a slight conformational change in these effector sites due to CaM binding could have significant impact on either the TRP domain or the coiled-coil domain or both, leading to distinct functional outcomes. The unique position of CaMBD thus not only enables TRPA1 to sense Ca^2+^, but also allows the channel to translate Ca^2+^ signals into channel gating. The revealed accessible CaMBD in TRPA1 could thus be a potential drug targeting region for tuning the channel for therapeutics.

## Materials and Methods

### Cell culture and transfection

HEK293 cells and mice DRG neurons were cultured as described previously^53^,^54^,^55^. They were maintained in DMEM medium containing 10% FBS supplemented with 100 U/ml penicillin, 100 μg/ml streptomycin and L-Glutamine (2 mM). HEK293 cells were transfected with either the PolyFect reagent (Qiagen) or the TurboFect reagent (Fermentus) as before^53^.

### Molecular biology

Thermo-TRP ion channels including TRPV1-4, TRPM8 (rat) and TRPA1 (mouse) cDNAs were subcloned into pcDNA3-V5-His-TOPO vector (Life Technology) as described previously^56^. Calmodulin (rat) cDNA was a kind gift of Dr. Ruth Lagnado (University of Sussex). CaM was added a HA tag to the N-terminus and subcloned into the pcDNA3.1 vector (Invitrogen) using standard PCR via KpnI and XbaI. The interleukin-2 receptor (Tac) cDNA was obtained from Dr. Juan Bonifacino (NIH, USA). To produce chimeric Tac-A1-CaMBD, the cytoplasmic tail in the C-terminus of Tac (T260–I272) was replaced by the corresponding C-terminus of mTRPA1 (G965-N1011) containing CaMBD via HinIII and XbaI using standard PCR. GST-coupled N- and C-terminus of TRPA1 were constructed by amplifying the corresponding cytoplasmic fragments (N-terminus: 1 M–R719; C-terminus: G965–F1125) followed by in-frame subcloning into a GST-pcDNA3 vector via BamHI and EcoRI. The GST-pcDNA3 vector was prepared by fusing the GST tag amplified from the pGEX-2T vector (GE healthcare) to the pcDNA3 vector (Invitrogen) via HindIII and BamHI. The Flag tag (DYKDDDDK) was also added to the C-terminus of cytoplasmic fragments of TRPA1 by PCR followed by subcloning into the pcDNA3.1 vector via KpnI and XbaI. To generate truncated GST-coupled C-terminus of TRPA1, a stop codon was introduced at relevant sites using the Quick-Change mutagenesis kit (Agilent Technologies). Quick-Change mutagenesis was also used to prepare all other mutations. We also employed Quick-Change mutagenesis to generate CaMBD-deleted TRPA1 using partially overlapping primers lacking the CaMBD region. All the constructs and mutations were validated by DNA sequencing.

### Pull down assay and co-immunoprecipitation

CaM pull down assay was performed by incubating CaM-agarose (Sigma) with HEK293 cell lysate expressing TRPA1-V5-His (×6) or other TRP channels in a lysis buffer (20 mM Tris-HCl, pH, 7.4, 150 mM NaCl, 1% NP-40 plus protease inhibitor cocktails (Roche)) at 4 °C under different Ca^2+^ or Ba^2+^ concentrations as indicated in related Figures. For CaM pull down assay in Fig. 3H and co-immunoprecipitation in Fig. 6E, TRPA1 and its mutants containing the 6×His tag expressed from HEK293 cells were first purified using Ni-NTA beads (Qiagen) as described previously^55^. After thorough washing, TRPA1 and mutant proteins were then eluted with 250 mM imidazole (Sigma). Equal amount of eluted TRPA1 proteins was then used for CaM pull down assay or co-immunoprecipitation. To obtain Ca^2+^-free, 0.5 mM EGTA was included to deplete background Ca^2+^ in the solution. Similarly in experiments for studying the effect of Ba^2+^, corresponding Ba^2+^ ions were added to the above Ca^2+^-free solution to exclude the effect of background Ca^2+^. For CaM pull down in Fig. 2C, free Ca^2+^ of 100 nM and 500 nM were obtained by adding Ca^2+^ (in mM) of 0.28 and 0.422, respectively, to the above Ca^2+^-free binding solution buffered by 0.5 mM EGTA. Free Ca^2+^ concentrations were calculated using the programme MaxChelator (University of Stanford). After 4 times of thorough washing (20 mins each) with corresponding free Ca^2+^, bound proteins were eluted by boiling in sample buffer followed by separation on a 7.5% SDS-PAGE gel and detection with anti-V5 (Life Technologies). A peptide (NH_2_-LWYLRKVDQRSTIVYPN-COOH) with identical sequence to CaMBD in TRPA1 and corresponding scrambled peptide (NH_2_-YNQIRVYKVTPRLSLDW-COOH) (Biomatik) were also included in CaM pull down assay to investigate the effect of peptide on TRPA1-CaM binding.

To examine the binding of CaM to cytoplasmic tails of TRPA1, GST-coupled TRPA1 cytoplasmic tails expressed from HEK293 cells were purified using GST-agarose (Sigma) followed by elution with Glutathione (10 mM), similarly as previously described^55^. Purified N- and C-terminus of TRPA1 coupled to GST were then incubated with CaM-agarose for CaM pull down assay. Bound GST-coupled TRPA1 fragments were then eluted with 100 mM EGTA followed by separation on a 10% SDS-PAGE gel and blot detection with anti-GST (GE Healthcare). Similarly, Flag-coupled N- and C-termini of TRPA1 expressed from HEK293 cells were first isolated using Flag-agarose (Sigma) and purified through thorough washing. Flag-pull down assay was then performed by incubating pure CaM (Sigma) with the isolated Flag-tagged TRPA1 fusion proteins bound to Flag agarose, followed by extensive washing and subsequent elution with the Flag peptide (Sigma). Eluted CaM was next analysed on 10% SDS-PAGE and detected by anti-CaM (Millipore). Flag-tagged fusion proteins were probed by anti-Flag (Sigma). Co-immunoprecipitation between CaM and TRPA1 was performed similarly as described previously^55^,^56^. Briefly, HA-CaM or related mutants was co-expressed with TRPA1-V5 in HEK293 cells followed by solubilisation. HA-CaM was then precipitated by monoclonal anti-HA.11 (Covance) and Protein A/G PLUS-Agarose (Santa Cruz Biotechnology). Co-precipitated TRPA1 was then resolved on a 10% SDS-PAGE gel followed by immunodetection with anti-V5. The band intensity of blots was quantified using Image J.

Silver staining of CaM-peptide complex was performed as described by others with slight modifications^52^. Briefly, 600 nM CaM was incubated with different molar ratios of peptides in a buffer containing 10 mM Na-HEPES (pH 7.2) and 2 mM Ca^2+^ or without Ca^2+^ (5 mM EGTA) at room temperature for 1 h. The formed protein complexes were then resolved on 12% non-denaturing polyacrylamide gels in the presence of 2 mM Ca^2+^ or without Ca^2+^ (2 mM EGTA) followed by staining with a silver kit (Sigma) in accordance with the manufacturer’s instructions.

### Electrophysiology

Whole-cell recordings were conducted using Axopatch 200B patch clamp amplifier (Axon) controlled by pClampx 10.2 softwares (Molecular Device) as before^55^,^56^. The basic extracellular solution (nominal 0 Ca^2+^) consisted of (in mM): 140 NaCl, 4 KCl, 1.0 MgCl_2_, 10 HEPES, 5 Glucose, pH 7.4 with NaOH. Ca^2+^-free solution was prepared by adding 5 mM EGTA to the basic extracellular solution. Solutions with millimolar free Ca^2+^ or Ba^2+^ were obtained by adding Ca^2+^ or Ba^2+^ directly to the basic extracellular solution. Internal pipette solution contained (in mM): 140 KCl, 2.0 MgCl_2_, 5.0 EGTA, 10 HEPES, pH 7.4 with KOH. To obtain 10 μM free Ca^2+^, 1.99 mM Ca^2+^ was added to a Ca^2+^-free solution buffered by 2 mM EGTA. To study the effect of the CaM antagonist W-7, cells were perfused with 100 μM W-7 (Tocris) throughout the recording. To study the effect of peptides on TRPA1 currents in DRG neuros, peptides were included in the pipette to dialyse the cells as described previously^56^. All the recordings were held at −60 mV and signals were analogue filtered using a 1 kHz low-pass Bessel filter. Time constants (τ) of desensitization induced by AITC were determined by fitting TRPA1 peak currents with the equation y = A * exp (−x/τ) + C using the Clampfit 10.2 software (Molecular Devices).

### Statistics

All data were presented as mean ± SEM. Differences between two groups were determined using *Student*’*s t* test and was considered significant if *P* < 0.05. Comparisons among multiple groups were assessed by one-way analysis of variance with Bonferroni’s *post hoc* test.

## Additional Information

**How to cite this article:** Hasan, R. *et al*. Calmodulin is responsible for Ca^2+^-dependent regulation of TRPA1 Channels. *Sci. Rep.* **7**, 45098; doi: 10.1038/srep45098 (2017).

**Publisher's note:** Springer Nature remains neutral with regard to jurisdictional claims in published maps and institutional affiliations.
