# Corrigendum: Calmodulin is responsible for Ca^2+^-dependent regulation of TRPA1 Channels

**DOI:** 10.1038/srep46588

**Published:** 2017-05-04

**Authors:** Raquibul Hasan, Alasdair T. S. Leeson-Payne, Jonathan H. Jaggar, Xuming Zhang

Scientific Reports 7: Article number: 4509810.1038/srep45098; published online: 03 23 2017; updated: 07 04 2015

The Acknowledgements section in this Article is incomplete.

“RH was supported by a PhD studentship from the Islamic Development Bank and the Cambridge Commonwealth Trust. ATSLP was supported by a BBSRC EastBio Ph. D studentship. This work was partly funded by an MRC new investigator research grant (to XZ) and a Royal Society project grant (to XZ).”

Should read:

“RH was supported by a PhD studentship from the Islamic Development Bank and the Cambridge Commonwealth Trust. ATSLP was supported by a BBSRC EastBio Ph. D studentship. This work was partly funded by an MRC new investigator research grant (to XZ) and a Royal Society project grant (to XZ).

To access the underlying data for this publication please see http://doi.org/10.17036/researchdata.aston.ac.uk.00000218”.

